# Supplementary material for: Grippenet: A New Tool for the Monitoring, Risk-Factor and Vaccination Coverage Analysis of Influenza-Like Illness in Switzerland
Source: Vaccines (Basel). 2020 Jun 27;8(3):343. doi: 10.3390/vaccines8030343 (PMC7565003; doi:10.3390/vaccines8030343)
Supplement: Supplementary file 1 [file vaccines-08-00343-s001.zip › vaccines-805609-supplementary/S1-Intake questionnaire.docx]

**Intake questionnaire – English**

* **For whom are you filling in this survey?**

- A member of my household
- Myself
- Someone else


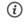


* **What is your gender?**

- Male
- Female


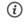


* **What is your date of birth (month and year)?**

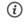


* **What is the first part of your home postcode (the part before the space)?**

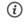


* **What is your main activity?**

- Paid employment, full time
- Paid employment, part time
- Self-employed (businessman, farmer, tradesman, etc.)
- Attending daycare/school/college/university
- Home-maker (e.g. housewife)
- Unemployed
- Long-term sick-leave or parental leave
- Retired
- Other


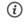


* **Except people you meet on public transports, do you have contact with any of the following during the course of a typical day? (Select all options that apply, if any)**

- More than 10 children or teenagers over the course of the day
- More than 10 people aged over 65 over the course of day
- Patients
- Groups of people (more than 10 individuals at any one time)
- None of the above


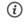


**INCLUDING YOU, how many people in each of the following age groups live in your household?**

- 0-4 years
- 5-18 years
- 19-44 years
- 45-64 years
- 65+

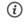


* **What is your main means of transport?**

- Walking
- Bike
- Motorbike/scooter
- Car
- Public transportation (bus, train, tube, etc)
- Other


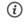


* **On a normal day, how much time do you spend on public transport? (Bus, train, tube etc.)**

- No time at all
- 0-30 minutes
- 30 minutes - 1.5 hours
- 1.5 hours - 4 hours
- Over 4 hours

**How often do you have common colds or flu-like diseases?**

- Never
- Once or twice a year
- Between 3 and 5 times a year
- Between 6 and 10 times a year
- More that 10 times a year
- I don't know


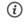


* **Did you receive a flu vaccine during the last autumn/winter season? (2016-2017)**

- Yes
- No
- I don't know


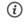


* **Have you received a flu vaccine this autumn/winter season? (2017-2018)**

- Yes
- No
- I don't know


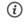


* **Do you take regular medication for any of the following medical conditions? (Select all options that apply)**

- No
- Asthma
- Diabetes
- Chronic lung disorder besides asthma e.g. COPD, emphysema, or other disorders that affect your breathing
- Heart disorder
- Kidney disorder
- An immunocompromising condition (e.g. splenectomy, organ transplant, acquired immune deficiency, cancer treatment)


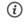


* **Do you smoke tobacco?**

- No
- Yes, occasionally
- Yes, daily, fewer than 10 times a day
- Yes, daily, 10 or more times a day
- Dont know/would rather not answer


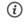


* **Do you have one of the following allergies that can cause respiratory symptoms? (Select all options that apply)**

- Hay fever
- Allergy against house dust mite
- Allergy against domestic animals or pets
- Other allergies that cause respiratory symptoms (e.g. sneezing, runny eyes)
- I do not have an allergy that causes respiratory symptoms

**Do you follow a special diet? (Select all options that apply)**

- No special diet
- Vegetarian
- Veganism
- Low-calorie
- Other

**Do you have pets at home? (Select all options that apply)**

- No
- Yes, one or more dogs
- Yes, one or more cats
- Yes, one or more birds
- Yes, one ore more other animals

**Where did you first hear about the flusurvey?**

- On radio or television
- In the newspaper or in a magazine
- Via an internet site (search engine or link)
- By poster
- Via family or friends
- Via school or work
