# Supplementary material for: Grippenet: A New Tool for the Monitoring, Risk-Factor and Vaccination Coverage Analysis of Influenza-Like Illness in Switzerland
Source: Vaccines (Basel). 2020 Jun 27;8(3):343. doi: 10.3390/vaccines8030343 (PMC7565003; doi:10.3390/vaccines8030343)
Supplement: Supplementary file 1 [file vaccines-08-00343-s001.zip › vaccines-805609-supplementary/S2-weekly questionnaire.docx]

**Symptoms questionnaire:**

**Q1 (if yes to any of these options except the first, continue with Q2)**

**Have you had any of the following symptoms since your last visit (or in the past week, if this is your first visit)?**

(Select all options that apply)

- No symptoms 0
- Fever 1
- Chills 2
- Runny or blocked nose 3
- Sneezing 4
- Sore throat 5
- Cough 6
- Shortness of breath 7
- Headache 8
- Muscle/joint pain 9
- Chest pain 10
- Feeling tired or exhausted (malaise) 11
- Loss of appetite 12
- Coloured sputum/phlegm 13
- Watery, bloodshot eyes 14
- Nausea 15
- Vomiting 16
- Diarrhoea 17
- Stomach ache 18
- Loss of smell 20
- Loss of taste 21
- Nose bleed 22
- Other 19

**Q2 (**(If the participant was STILL ILL on their last visit and has reported symptoms this time, otherwise this question remains hidden):

“On DATE OF LAST VISIT you reported that you were still ill with symptoms that began on DATE OF FIRST SYMPTOMS REPORTED PREVIOUSLY.

**Are the symptoms you reported today part of the same bout of illness?**

- Yes
- No
- I don’t know/can’t remember

**Q3 (if symptoms)**

**When did the first symptoms appear?**

- Choose date XX/XX/XXXX
- I don’t know/can’t remember

**Q4 (if symptoms)**

**When did your symptoms end?**

- Choose date XX/XX/XXXX
- I don’t know/can’t remember
- I am still ill

**Q5 (if symptoms)**

**Did your symptoms develop suddenly over a few hours?**

- Yes
- No
- I don’t know/can’t remember

**Weekly Q6 (if fever)**

**When did your fever begin?**

- Choose date XX/XX/XXXX
- I don’t know/can’t remember

**Q6b (extra non-core question) (if fever)**

**Did your fever develop suddenly over a few hours?**

- Yes
- No
- Don't know

**Q6c (if symptoms)**

**Did you take your temperature?**

- Yes [go to Weekly Q6c]
- No
- I don’t know

**Q6d (if symptoms) and (if took temperature): follow-up question**

**What was your highest temperature measured?**

- Below 37° C
- 37° - 37.4°C
- 37.5° - 37.9°C
- 38° – 38.9°C
- 39° - 39.9°C
- 40°C or more
- I don’t know/can’t remember

**Q7 (if symptoms)**

**Because of your symptoms, did you VISIT (see face to face or at home) any of medical services?**

(Select all options that apply)

- No
- GP or GP’s practice nurse
- Hospital admission
- Hospital accident & emergency department/out of hours service
- Other medical services
- No, but I have an appointment scheduled

**Q7b (if symptoms)**

**How soon after your symptoms appeared did you first visit this medical service?**

- Same day
- 1 day
- 2 days
- 3 days
- 4 days
- 5-7 days
- More than 7 days
- I don’t know/can’t remember

**Q8 (if symptoms)**

**Because of your symptoms, did you contact via TELEPHONE or INTERNET (video or teleconsultation) any of the following?**

(Select all options that apply)

- No
- GP – spoke to receptionist only
- GP – spoke to doctor or nurse
- NHS Direct / NHS 24 / NHS Choices
- NPFS
- Other

**Q8b (if symptoms)**

**How soon after your symptoms appeared did you first contact via telephone or internet any of the services?**

- Same day
- 1 day
- 2 days
- 3 days
- 4 days
- 5-7 days
- More than 7 days
- I don’t know/can’t remember

**Q9 (if symptoms)**

**Did you take medication for these symptoms?**

(Select all options that apply)

- No medication 0
- Pain killers (e.g. paracetamol, aspirin, ibuprofen, etc) 1
- Cough medication (e.g. expectorants) 2
- Antivirals (Tamiflu, Relenza) 3
- Antibiotics 4
- Homeopathy 7
- Alternative medicine (essential oil, phytotherapy, etc.) 8
- Other 5
- I don’t know/can’t remember 6

**Q9b (if antivirals were taken): follow-up question**

**How long after the beginning of your symptoms did you start taking antiviral medication?**

- Same day (within 24 hours)
- 1 day later
- 2 days later
- 3 days later
- 4 days later
- 5-7 days later
- More than 7 days later
- I don’t know/can’t remember

**Q14 (if symptoms)**

**Because of your symptoms, were you hospitalized?**

•Yes 1
• No 0

**Q10 (if symptoms)**

**Did you change your daily routine because of your illness?**

- No
- Yes, but I did not take time off work/school
- Yes, I took time off work/school

**Q10b (if symptoms) & (if taken time off work/school): follow-up question**

**Are you still off work/school?**

- Yes
- No
- Other (e.g. I wouldn’t usually be at work/school today anyway)

**Q10c (if symptoms) & (if taken time off work/school): follow-up question**

How have you been off work/school for?

- 1 day
- 2 days
- 3 days
- 4 days
- 5 days
- 6 to 10 days
- 11 to 15 days
- More than 15 days

**Q11 (if symptoms)**

**What do you think is causing your symptoms?**

- Flu or flu-like illness 0
- Common cold 1
- Allergy/hay fever 2
- Asthma 6
- Gastroenteritis/gastric flu 3
- New coronavirus (Covid-19) 9
- Other 4
- I don’t know 5
